# Tumor extracellular vesicles mediate anti-PD-L1 therapy resistance by decoying anti-PD-L1

**DOI:** 10.1038/s41423-022-00926-6

**Published:** 2022-10-11

**Authors:** Jiming Chen, Jie Yang, Wenhui Wang, Danfeng Guo, Chengyan Zhang, Shibo Wang, Xinliang Lu, Xiaofang Huang, Pingli Wang, Gensheng Zhang, Jing Zhang, Jianli Wang, Zhijian Cai

**Affiliations:** 1 Institute of Immunology, and Department of Orthopaedics of the Second Affiliated Hospital, Zhejiang University School of Medicine, 310058 Hangzhou, China; 2 Henan Key Laboratory for Digestive Organ Transplantation, The First Affiliated Hospital of Zhengzhou University, 450052 Zhengzhou, China; 3 Department of Critical Care Medicine, Qilu Hospital, Cheeloo College of Medicine, Shandong University, 250063 Jinan, China; 4 Department of Respiratory and Critical Care Medicine, The Second Affiliated Hospital of Zhejiang University School of Medicine, 310003 Hangzhou, China; 5 Department of Critical Care Medicine of the Second Affiliated Hospital, Zhejiang University School of Medicine, 310003 Hangzhou, China; 6 Department of Pathology, Zhejiang University First Affiliated Hospital and School of Medicine, 310002 Hangzhou, China; 7 Institute of Immunology, and Bone Marrow Transplantation Center of the First Affiliated Hospital, Zhejiang University School of Medicine, 310058 Hangzhou, China; 8 Institute of Hematology, Zhejiang University & Zhejiang Engineering Laboratory for Stem Cell and Immunotherapy, 310006 Hangzhou, China

**Keywords:** Tumor, Extracellular vesicles, PD-L1, Immunotherapy, Tumour immunology

## Abstract

PD-L1^+^ tumor-derived extracellular vesicles (TEVs) cause systemic immunosuppression and possibly resistance to anti-PD-L1 antibody (αPD-L1) blockade. However, whether and how PD-L1^+^ TEVs mediate αPD-L1 therapy resistance is unknown. Here, we show that PD-L1^+^ TEVs substantially decoy αPD-L1 and that TEV-bound αPD-L1 is more rapidly cleared by macrophages, causing insufficient blockade of tumor PD-L1 and subsequent αPD-L1 therapy resistance. Inhibition of endogenous production of TEVs by Rab27a or Coro1a knockout reverses αPD-L1 therapy resistance. Either an increased αPD-L1 dose or macrophage depletion mediated by the clinical drug pexidartinib abolishes αPD-L1 therapy resistance. Moreover, in the treatment cycle with the same total treatment dose of αPD-L1, high-dose and low-frequency treatment had better antitumor effects than low-dose and high-frequency treatment, induced stronger antitumor immune memory, and eliminated αPD-L1 therapy resistance. Notably, in humanized immune system mice with human xenograft tumors, both increased αPD-L1 dose and high-dose and low-frequency treatment enhanced the antitumor effects of αPD-L1. Furthermore, increased doses of αPD-L1 and αPD-1 had comparable antitumor effects, but αPD-L1 amplified fewer PD-1^+^ Treg cells, which are responsible for tumor hyperprogression. Altogether, our results reveal a TEV-mediated mechanism of αPD-L1-specific therapy resistance, thus providing promising strategies to improve αPD-L1 efficacy.

## Introduction

The application of immune checkpoint blockade, including anti-PD-1 and anti-PD-L1 antibodies (αPD-1 and αPD-L1), has led to a major revolution in tumor immunotherapy. Although αPD-1 and αPD-L1 show excellent efficacy in various tumor types, even in patients with advanced tumors [1–3], only 10–30% of patients respond to αPD-1 and αPD-L1 therapy due to primary resistance [4, 5]. In addition, some patients who initially respond to αPD-1 and αPD-L1 therapy eventually acquire resistance, leading to disease progression [4, 6]. Loss of β2-microglobulin in tumor cells contributes to αPD-1- and αPD-L1-therapy resistance [7]. Defects in the interferon signaling pathway of tumor cells have also been proposed as a potential mechanism for αPD-1- and αPD-L1 therapy resistance [8]. However, whether there are distinct mechanisms responsible for αPD-1 and αPD-L1 therapy resistance remains unknown.

Extracellular vesicles (EVs) are mainly divided into two categories: ectosomes and exosomes. Ectosomes (50–1000 nm in diameter) are vesicles produced by direct outward budding of the plasma membrane. Exosomes (30–150 nm in diameter) are generated from the endosomal pathway. EVs contain large numbers of proteins, nucleic acids, lipids and metabolites from their parent cells and are essential for communication between cells [9]. PD-L1 has been reported to occur on tumor-derived EVs (TEVs), and TEV PD-L1 plays a central role in the induction of immune escape [10]. PD-L1 on melanoma-derived EVs inhibits the activation of CD8^+^ T cells and facilitates tumor growth [11]. TEV PD-L1 induces systemic immunosuppression and appears to be resistant to αPD-L1 therapy [12]. TEV PD-L1 is related to immunotherapy resistance, and inhibition of TEV secretion greatly enhanced the efficiency of αPD-L1 therapy in a 4T1 breast tumor model [13]. These findings suggest that TEV PD-L1 is probably responsible for resistance to αPD-L1 therapy. However, the specific resistance mechanisms mediated by TEV PD-L1 are still unclear. Two secreted PD-L1 splicing variants that lack the transmembrane domain have been demonstrated to act as “decoys” for αPD-L1, thereby causing αPD-L1 therapy resistance [14]. Similarly, in addition to the transduction of inhibitory signaling by binding PD-1 on T cells, whether TEV PD-L1 may also decoy αPD-L1, resulting in the consumption of αPD-L1 and consequent therapy resistance, is currently unclear.

PD-1 has two naturally occurring ligands, PD-L1 and PD-L2, that provide inhibitory signals to T cells via PD-1 [15]. αPD-1 blocks the inhibitory signal triggered by both PD-L1 and PD-L2, while αPD-L1 interrupts only immunosuppression mediated by PD-L1. Theoretically, the antitumor effect of αPD-1 is expected to be better than that of αPD-L1. However, there is still no proof-of-principle study comparing the effects of αPD-1 and αPD-L1 on tumor therapy. Furthermore, there is no metric to predict whether a patient will benefit more from αPD-1 or αPD-L1 therapy. Circulating TEV PD-L1 increases with tumor progression [16], which consumes large amounts of αPD-L1 but not αPD-1. Thus, TEV PD-L1 probably weakens the therapeutic effects of αPD-L1, and circulating TEV PD-L1 may be a useful metric for predicting the outcome of αPD-1 and αPD-L1 therapy, which has yet to be explored.

Here, we found that TEVs can efficiently decoy αPD-L1 via PD-L1. TEV-bound αPD-L1 is more readily phagocytized by macrophages and then more rapidly degraded by lysosomes. In this way, TEVs consume large amounts of αPD-L1, leading to insufficient αPD-L1 to block PD-L1 on tumor cells, thereby mediating αPD-L1 therapy resistance.

## Results

### TEV PD-L1 competes with PD-L1 on tumor cells to bind αPD-L1

**Fig. 1:**
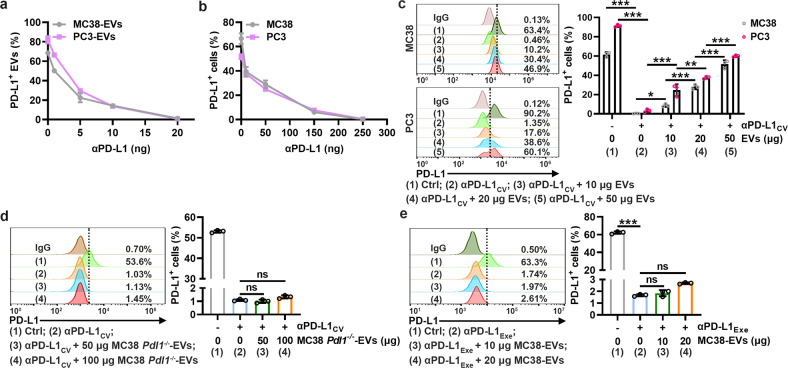
TEV PD-L1 competes with PD-L1 on tumor cells to bind αPD-L1. **a**, **b** MC38-EVs and PC3-EVs (1 μg) (**a**) or MC38 and PC3 cells (1 × 10^5^) (**b**) were coincubated with the indicated doses of αPD-L1 in 100 μl of medium for 30 min. Then, PD-L1 on EVs (**a**) or cells (**b**) was detected by flow cytometry. **c** A total of 1 × 10^5^ MC38 and PC3 cells were coincubated with αPD-L1_CV_ with or without the corresponding EVs at the indicated doses in 100 μl of medium for 30 min. Then, PD-L1 on the cells was detected by flow cytometry. **d**, **e** A total of 1 × 10^5^ MC38 cells were coincubated with αPD-L1_CV_ (**d**) or αPD-L1_Exe_ (**e**) in the presence of the indicated doses of MC38 *Pdl1*^*−/−*^-EVs (**d**) or MC38-EVs (**e**) in 100 μl of medium for 30 min. Then, PD-L1 on MC38 cells was detected by flow cytometry. The αPD-L1 for coincubation and detection recognizes the same epitope in PD-L1. Representative results from three independent experiments are shown (*n* = 3). *P < 0.05; **P < 0.01; ***P < 0.001; ns, not significant (one-way ANOVA followed by Tukey’s test; mean and s.d.)

To explore whether PD-L1 on TEVs can competitively bind αPD-L1 with PD-L1 on tumor cells, we isolated EVs from murine MC38 colon cancer cells (MC38-EVs) and human PC3 prostate cancer cells (PC3-EVs) that have been reported to contain high levels of PD-L1. These EVs showed typical exosome-like morphology (Supplementary Fig. 1a), contained CD63, Tsg101, Alix and CD81 but not GRP94 (Supplementary Fig. 1b), and had a mean size of 198 ± 88 nm for MC38-EVs and 193 ± 69 nm for PC3-EVs (Supplementary Fig. 1c). As expected, we detected high levels of total and membrane PD-L1 on both EVs (Supplementary Fig. 1b, d), and with increasing αPD-L1 coincubated with MC38-EVs and PC3-EVs, decreased αPD-L1-free PD-L1 proteins on both EVs were detected (Supplementary Fig. 1e), indicating the binding of αPD-L1 and PD-L1 on EVs. The maximal binding amount of αPD-L1 by 1 μg MC38-EVs and PC3-EVs was approximately 20 ng (Fig. 1a). In addition, we confirmed that the minimal amount of αPD-L1 (critical value of αPD-L1, αPD-L1_CV_) that occupied all PD-L1 on 1 × 10^5^ MC38 and PC3 cells was approximately 250 ng (Fig. 1b and Supplementary Fig. 1f). At αPD-L1_CV_, the addition of MC38-EVs and PC3-EVs dose-dependently increased αPD-L1-free PD-L1 on MC38 and PC3 cells (Fig. 1c). However, EVs from MC38 cells with PD-L1 knockout (MC38 *Pdl1*^*−/−*^-EVs) did not affect the binding of αPD-L1 and PD-L1 to MC38 cells (Fig. 1d and Supplementary Fig. 1g). Furthermore, when excess αPD-L1 (αPD-L1_Exe_) was used to block PD-L1 on MC38 cells, the addition of MC38-EVs no longer increased the αPD-L1-free PD-L1 on MC38 cells (Fig. 1e). The method used to isolate TEVs in this study is the classical protocol for exosome-like vesicle concentration, and TEVs isolated by this method have a relatively small size. Therefore, we also isolated microvesicles from MC38 and PC3 cells (MC38-MVs and PC3-MVs) and confirmed that they both contained vesicles larger than 200 nm in diameter (Supplementary Fig. 1h). We found that MC38-MVs and PC3-MVs also carried membrane-associated PD-L1 and increased αPD-L1-free PD-L1 on MC38 and PC3 cells at αPD-L1_CV_ (Supplementary Fig. 1i, j). Altogether, these results indicate that TEV PD-L1 competes with tumor PD-L1 to bind αPD-L1.

### TEVs impair αPD-L1-induced CD8^+^ T-cell proliferation by decoying αPD-L1

**Fig. 2:**
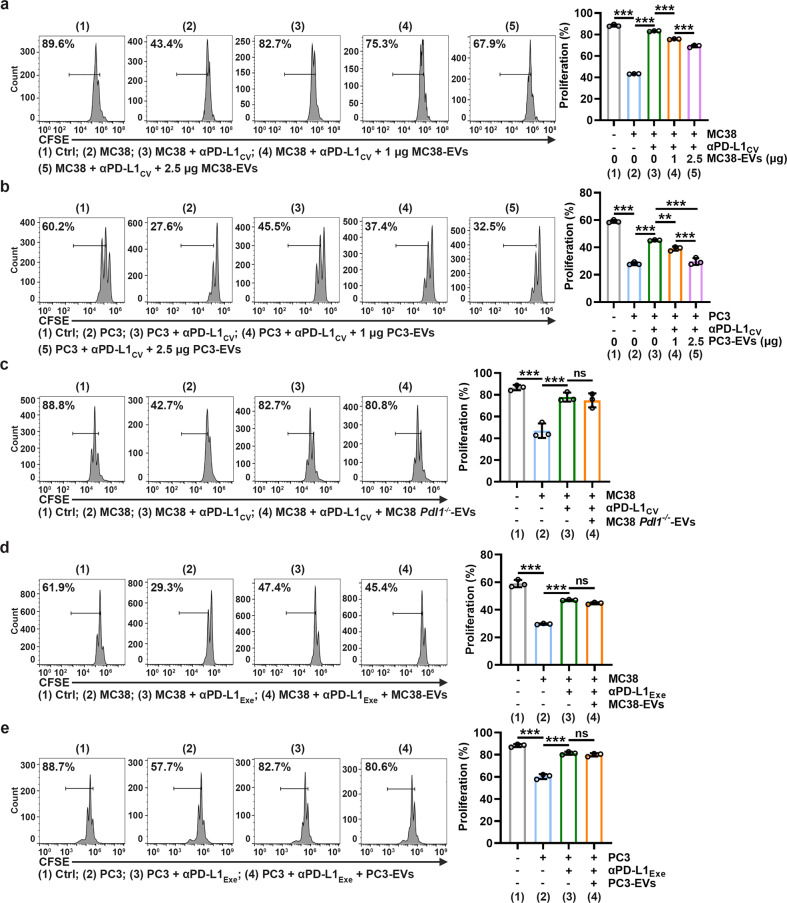
TEVs impair αPD-L1-induced CD8^+^ T-cell proliferation by decoying αPD-L1. **a**, **b** CFSE-labeled CD8^+^ T cells were stimulated with 2 μg ml^−1^ anti-CD3 and anti-CD28 for 24 h and then coincubated with 5 × 10^4^ MC38 (**a**) or PC3 (**b**) cells, αPD-L1_CV_ with or without the indicated doses of MC38-EVs (**a**) or PC3-EVs (**b**) in 200 μl of medium for 48 h. Then, the CFSE dilution was measured by flow cytometry. **c**–**e** CFSE-labeled CD8^+^ T cells were stimulated with 2 μg ml^-1^ anti-CD3 and anti-CD28 for 24 h and then coincubated with 5 × 10^4^ MC38 (**c**, **d**) or PC3 (**e**) cells, αPD-L1_CV_ (**c**) or αPD-L1_Exe_ (**d**, **e**) with or without 2.5 μg of MC38 *Pdl1*^*−/−*^-EVs (**c**), MC38-EVs (**d**) or PC3-EVs (**e**) in 200 μl of medium for 48 h. Then, the CFSE dilution was measured by flow cytometry. Representative results from three independent experiments are shown (*n* = 3). ***P < 0.001 (one-way ANOVA followed by Tukey’s test; mean and s.d.)

Blockade of PD-L1 on tumor cells by αPD-L1 normalizes antitumor CD8^+^ T-cell responses [17]. Since TEVs compete with tumor PD-L1 to bind αPD-L1, we then investigated whether TEVs can prevent the normalization of CD8^+^ T-cell responses by consuming αPD-L1. As expected, PD-L1^+^ MC38 cells inhibited anti-CD3/CD28-induced CD8^+^ T-cell proliferation, which was eliminated by αPD-L1_CV_ (Fig. 2a). However, the addition of MC38-EVs dose-dependently restored the MC38 cell-mediated proliferative inhibition of CD8^+^ T cells (Fig. 2a). Similar results were obtained in the PC3 cell and PC3-EV coculture system (Fig. 2b). Although MC38-EVs and PC3-EVs were positive for PD-L1, none of them inhibited CD8^+^ T-cell proliferation alone at the concentration we used (Supplementary Fig. 2a, b). In addition, TEVs specifically blunted the effect of αPD-L1 but not αPD-1 because MC38-EVs did not affect αPD-1-normalized CD8^+^ T-cell proliferation (Supplementary Fig. 2c), probably due to the absence of PD-1 on MC38-EVs (Supplementary Fig. 2d). Furthermore, MC38 *Pdl1*^*−/−*^-EVs were unable to affect αPD-L1 to rescue MC38 cell-mediated proliferative inhibition of CD8^+^ T cells (Fig. 2c), suggesting that PD-L1 on EVs is indispensable for this process. To further confirm the consumption of αPD-L1, we used αPD-L1_Exe_ to rescue CD8^+^ T-cell proliferation that was inhibited by PD-L1 on MC38 cells or PC3 cells. Under this condition, neither MC38-EVs nor PC3-EVs affected αPD-L1-mediated rescue of CD8^+^ T-cell proliferation (Fig. 2d, e). These results demonstrate that PD-L1 on TEVs consumes αPD-L1, leading to insufficient neutralization of PD-L1 on tumor cells by αPD-L1.

### TEV-mediated αPD-L1 consumption blunts the antitumor effect of αPD-L1

**Fig. 3:**
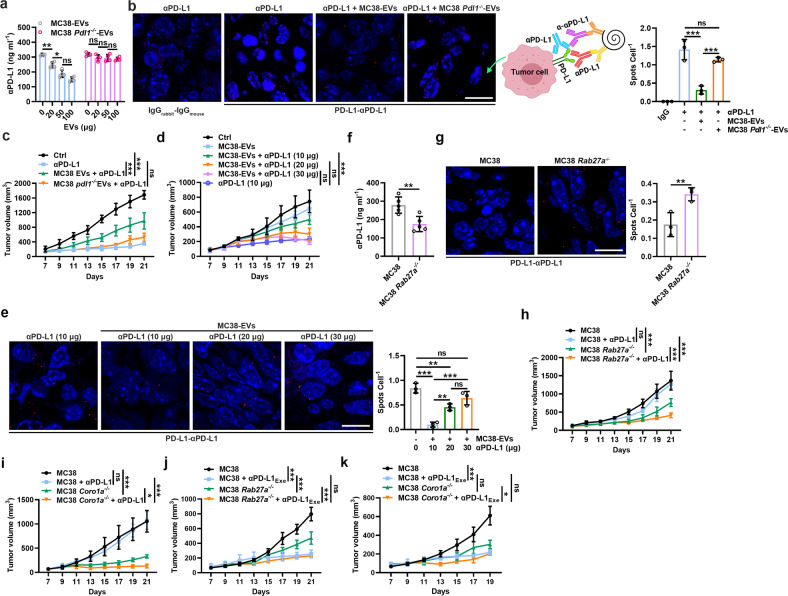
TEV-mediated αPD-L1 consumption blunts the antitumor effect of αPD-L1. **a**–**c** Mice with MC38 tumors were intravenously injected with 10 μg of αPD-L1 with or without the indicated doses (**a**) or with 20 μg (**b**, **c**) of MC38-EVs or MC38 *Pdl1*^*−/−*^-EVs every 2 days starting when the tumor size reached 100–200 mm^3^. PD-L1-free αPD-L1 levels in sera were measured by ELISAs 2 h after the first treatment (**a**), the interaction of αPD-L1 and tumor PD-L1 was detected by PLA on Day 21 (**b**), and the tumor sizes were monitored every other day (**c**). **d**, **e** Mice with MC38 tumors were intravenously injected with the indicated doses of αPD-L1 with or without 20 μg of MC38-EVs every 2 days starting when the tumor size reached 100–200 mm^3^. Tumor sizes were monitored every other day (**d**), and the interaction of αPD-L1 and tumor PD-L1 was detected by PLA on Day 21 (**e**). **f** PD-L1-free αPD-L1 levels in the sera of the mice with MC38 or MC38 *Rab27a*^*−/−*^ tumors were measured by ELISAs on Day 7. **g**–**k** Mice with MC38, MC38 *Rab27a*^*−/−*^ (**g**, **h**, **j**) or MC38 *Coro1a*^*−/−*^ (**i**, **k**) tumors were intravenously injected with 3 μg of αPD-L1 (**g**–**i**) or αPD-L1_Exe_ (**j**, **k**) every 2 days starting when the tumor size reached 100–200 mm^3^. The interaction of αPD-L1 and tumor PD-L1 was detected by PLA on Day 21 (**g**), and the tumor sizes were monitored every other day (**h**–**k**). Scale bar, 10 μm. Representative results from two independent experiments are shown (*n* = 3 in **a**, **b**, **e**–**g**; *n* = 5 in **c**, **d**, **h**–**k**). *P < 0.05; **P < 0.01; ***P < 0.001; ns not significant (one-way ANOVA followed by Tukey’s test except for unpaired two-tailed Student’s *t* test in **f**, **g**; mean and s.d.)

Then, we examined whether TEVs can consume αPD-L1 in vivo. We first confirmed that circulating EVs (Circ-EVs) from mice with 1-, 2- and 4-week MC38 tumors bound approximately 0.04 ± 0.02, 0.12 ± 0.01 and 0.16 ± 0.01 μg (mean ± s.d.; *n* = 3) of αPD-L1, respectively. However, EVs from tumor tissues (EVs-TT) of these mice bound approximately 0.95 ± 0.20, 1.69 ± 0.16 and 2.51 ± 0.14 μg (mean ± s.d.; *n* = 3) of αPD-L1, respectively, which was remarkably high. In addition, the PD-L1 levels of Circ-EVs were positively correlated with those of EVs-TT (Supplementary Fig. 3a), as was the amount of αPD-L1 bound by Circ-EVs and EVs-TT (Supplementary Fig. 3b). Subsequently, we administered αPD-L1 and MC38-EVs to MC38 tumor-bearing mice. We found that MC38-EVs dose-dependently reduced PD-L1-free αPD-L1 levels in serum, which could not be achieved by MC38 *Pdl1*^*−/−*^-EVs (Fig. 3a). When αPD-L1 and tumor PD-L1 interactions were detected by a proximity ligation assay (PLA), we found that the PLA spots on tumor cells were obviously reduced by MC38-EVs but not MC38 *Pdl1*^*−/−*^-EVs (Fig. 3b), suggesting that MC38-EV PD-L1 and tumor PD-L1 competitively bound αPD-L1 in vivo. Consistent with these results, MC38-EVs but not MC38 *Pdl1*^*−/−*^-EVs greatly attenuated the antitumor effect of αPD-L1 (10 μg per injection) along with the decreased IFN-γ^+^CD8^+^ and Ki-67^+^CD8^+^ T cells in the TTs of the mice that received MC38-EVs but not MC38 *Pdl1*^*−/−*^-EVs (Fig. 3c and Supplementary Fig. 3c). At the dose we used, neither MC38-EVs nor MC38 *Pdl1*^*−/−*^-EVs promoted tumor growth, suggesting that MC38-EVs indeed blunted the antitumor effect of αPD-L1 by consuming αPD-L1 rather than directly inhibiting antitumor immunity (Supplementary Fig. 3d). To further confirm this, we injected serial doses of αPD-L1 and found that MC38-EVs ceased to impair the antitumor effect of αPD-L1 with enhanced αPD-L1 doses (Fig. 3d). Correspondingly, the αPD-L1 and tumor PD-L1 interaction increased with increasing αPD-L1 dose (Fig. 3e). In addition, the αPD-L1 dose did not affect the PD-L1 levels on TEVs (Supplementary Fig. 3e), but an increased αPD-L1 dose did reduce αPD-L1-free PD-L1 on TEVs (Supplementary Fig. 3f). These results suggest that an increased αPD-L1 dose also increases the TEV PD-L1 and αPD-L1 interaction.

αPD-L1 could bind to EVs from MC38 TTs but not MC38 *Pdl1*^*−/−*^ TTs, confirming the binding of endogenous TEV PD-L1 with αPD-L1 (Supplementary Fig. 3g). To evaluate the effect of endogenous TEVs on αPD-L1 antitumor activity, we used Rab27a-deficient MC38 cells (MC38 *Rab27a*^*−/−*^) with impaired TEV secretion (Supplementary Fig. 3h, i) to establish tumor-bearing mice. First, we confirmed that Rab27a deficiency did not affect the PD-L1 level on MC38 cells (Supplementary Fig. 3j). Unexpectedly, before an obvious difference in tumor size was observed, the serum levels of αPD-L1 from the MC38 *Rab27a*^*−/−*^ tumor-bearing mice were sharply lower than those from the MC38 tumor-bearing mice (Fig. 3f), probably due to the increased binding of circulating αPD-L1 to tumor PD-L1 during tumor development. We observed more αPD-L1 and MC38 *Rab27a*^*−/−*^ tumor PD-L1 interactions (Fig. 3g). A dose of αPD-L1 (3 μg per injection) with no therapeutic effect on MC38 tumors nonetheless significantly inhibited MC38 *Rab27a*^*−/−*^ tumor growth, accompanied by a significant increase in IFN-γ^+^CD8^+^ and Ki-67^+^CD8^+^ T cells in TTs (Fig. 3h and Supplementary Fig. 3k). To exclude the possibility that these results might be caused by Rab27a knockout itself, we established tumors by using MC38 cells with *Coro1a* knockout (MC38 *Coro1a*^*−/−*^), which release reduced TEVs [18]. Similar to the results from the MC38 *Rab27a*^*−/−*^ tumor-bearing mice, αPD-L1 notably inhibited MC38 *Coro1a*^*−/−*^ but not MC38 tumor growth at the same dose (Fig. 3i). However, if αPD-L1_Exe_ (30 μg per injection) was used, the difference in growth between MC38 tumors and MC38 *Rab27a*^*−/−*^ or MC38 *Coro1a*^*−/−*^ tumors was completely eliminated (Fig. 3j, k). In summary, these results indicate that TEV PD-L1 consumes αPD-L1, blunting the antitumor effect of αPD-L1.

### High-dose and low-frequency treatment reverses αPD-L1 therapy resistance

**Fig. 4:**
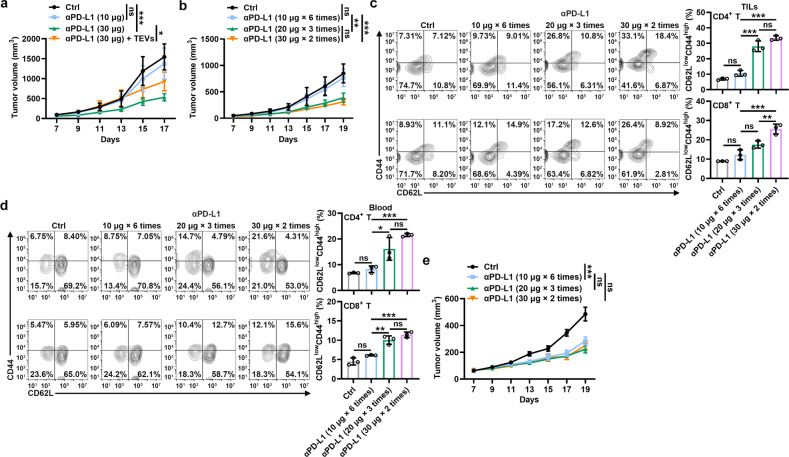
High-dose and low-frequency treatment reverses αPD-L1 therapy resistance. **a** Mice with TRAMP-C2 tumors were intravenously injected with the indicated doses of αPD-L1 with or without 20 μg of TRAMP-C2-EVs every 2 days when the tumor size reached 100–200 mm^3^. Tumor sizes were monitored every other day. **b**–**e** Mice with TRAMP-C2 (**b**, **d**) or TRAMP-C2 *Rab27a*^*−/−*^ (**e**) tumors were intravenously injected with αPD-L1 according to the indicated strategies every 2 days starting when the tumor size reached 100–200 mm^3^. Tumor sizes were monitored every other day (**b**, **e**). CD62L^low^CD44^high^ memory T cells in TILs (**c**) and blood (**d**) were analyzed by flow cytometry on Day 19 (**c**, **d**). Representative results from two independent experiments are shown (*n* = 5 in **a**, **b**, **e**; *n* = 3 in **c**, **d**). *P < 0.05; **P < 0.01; ***P < 0.001; ns not significant (one-way ANOVA followed by Tukey’s test; mean and s.d.)

Then, we wanted to determine whether excess consumption of αPD-L1 by TEVs leads to αPD-L1 therapy resistance. Because murine TRAMP-C2 prostate cancer has been proven to resist αPD-L1 blockade [19], we investigated the effect of αPD-L1_Exe_ treatment on TRAMP-C2 tumor progression. As expected, a low dose of αPD-L1 failed to inhibit TRAMP-C2 tumor growth, while a high dose showed successful inhibition (Fig. 4a). In addition, supplementation with TRAMP-C2-EVs significantly blunted the antitumor effect of a high dose of αPD-L1 (Fig. 4a). These results indicate that the consumption of αPD-L1 by TEVs is indeed involved in αPD-L1 therapy resistance. Given that there may be unknown risks of increasing the total therapeutic dose of αPD-L1, we treated tumor-carrying mice with high-dose and low-frequency αPD-L1 to keep the total dose of αPD-L1 unchanged throughout the treatment, supplying more TEV-free αPD-L1 for the blockade of tumor PD-L1 in each administration. We found that high-dose and low-frequency αPD-L1 treatment had notably stronger inhibitory effects on TRAMP-C2 tumor growth than low-dose and high-frequency αPD-L1 treatment (Fig. 4b). Corresponding to these results, enhanced memory CD4^+^ and CD8^+^ T cells in tumor infiltrating lymphocytes (TILs), peripheral blood and spleen were observed in the tumor-bearing mice receiving high-dose and low-frequency αPD-L1 treatment (Fig. 4c, d, and Supplementary Fig. 4a). Moreover, we obtained similar results in the MC38 tumor-bearing mice (Supplementary Fig. 4b, c). To validate the role of TEVs in this process, we performed the same experiments in the TRAMP-C2 *Rab27a*^*−/−*^ tumor-bearing mice and found that both treatments significantly inhibited tumor growth and had comparable antitumor effects after the production of endogenous TEVs was suppressed (Fig. 4e). Therefore, these results demonstrate that high-dose and low-frequency αPD-L1 treatment reverses TEV-mediated αPD-L1 therapy resistance by inducing stronger antitumor immune memory.

### Depletion of macrophages reverses αPD-L1 therapy resistance

**Fig. 5:**
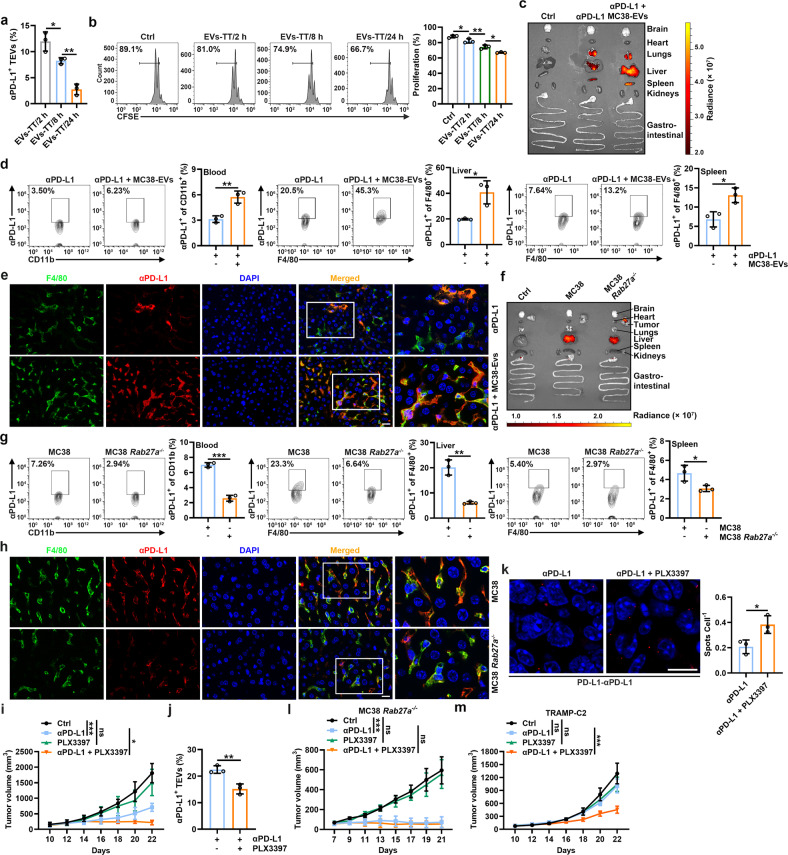
Depletion of macrophages reverses αPD-L1 therapy resistance. **a**, **b** Mice with MC38 tumors were intravenously injected with 10 μg of αPD-L1 for the indicated time. Then, αPD-L1-bound EVs-TT were detected (**a**), and the inhibitory effect of these EVs on CD8^+^ T-cell proliferation was assessed according to CFSE dilution (**b**) by flow cytometry. **c**–**h** Mice without tumors (**c**–**e**) or with MC38 or MC38 *Rab27a*^*−/−*^ tumors (**f**–**h**) were intravenously injected with 10 μg Alexa Fluor 680-labeled αPD-L1 with (**c**–**e**) or without (**f**–**h**) 20 μg MC38-EVs. The distribution of αPD-L1 was detected by an in vivo imaging system (IVIS) (**c**, **f**), αPD-L1 in blood monocytes and liver and spleen macrophages was detected by flow cytometry (**d**, **g**), and αPD-L1 in liver macrophages was detected by immunofluorescence (scale bar, 20 μm) (**e**, **h**) 24 h (**c**–**e**) or 21 days after tumor cell injection (**f**–**h**). **i**–**m** Mice with MC38 (**i**–**k**), MC38 *Rab27a*^*−/−*^ (**l**) or TRAMP-C2 (**m**) tumors were intravenously injected with 10 μg of αPD-L1 with or without intraperitoneal injection of 20 μg of PLX3397 every 2 days starting when the tumor size reached 100–200 mm^3^. Tumor sizes were monitored every other day (**i**, **l**, **m**). αPD-L1-bound EVs-TT were detected by flow cytometry (**j**), and the interaction of αPD-L1 and tumor PD-L1 was detected by PLA (scale bar, 10 μm) (**k**) on Day 22 (**j**, **k**). Representative results from two independent experiments are shown (*n* = 3 except for *n* = 5 in **i**, **l**, **m**). *P < 0.05; **P < 0.01; ***P < 0.001; ns not significant (one-way ANOVA followed by Tukey’s test in **a**, **b**, **i**, **l**, **m**; unpaired two-tailed Student’s *t* test in **d**, **g**, **j**, **k**; mean and s.d.)

PD-L1 on TEVs is involved in the inhibition of antitumor CD8^+^ T-cell responses [11, 12], so the blockade of PD-L1 on TEVs by αPD-L1 can also restrain the immunosuppressive function of TEVs. However, we found that αPD-L1-bound EV-TTs of the MC38 tumor-bearing mice treated with αPD-L1 decreased over time (Fig. 5a). Consistent with these results, the inhibitory effect of EVs from TTs on CD8^+^ T-cell proliferation in vitro increased over time (Fig. 5b). These results suggest that αPD-L1 might dissociate from TEVs over time. If so, the dissociated TEV-free αPD-L1 may bind tumor PD-L1, leading to the increased binding of αPD-L1 and tumor PD-L1 over time. However, we observed the opposite results (Supplementary Fig. 5a), which suggested that the increased αPD-L1-free TEVs over time were probably due to de novo TEVs rather than to the dissociation of αPD-L1 from TEVs. Therefore, we investigated the fate of TEV-bound αPD-L1. EVs have been reported to be cleared by monocytes, and transferred EVs accumulate predominantly in liver macrophages [20, 21]. When compared with free αPD-L1, enhanced MC38-EV-bound αPD-L1 was phagocytized by peritoneal macrophages (PMs) (Supplementary Fig. 5b). In addition, we found that MC38-EV-bound αPD-L1 tended to be transported into lysosomes (Supplementary Fig. 5c). These results suggest that EV binding promotes αPD-L1 degradation by macrophages. Next, we determined whether TEVs affect the fate of αPD-L1 in vivo. We injected αPD-L1 with or without MC38-EVs into tumor-free mice and found that when injected alone, αPD-L1 localized mainly in the lungs, followed by the liver and spleen (Fig. 5c). However, combined injection with MC38-EVs greatly enhanced the accumulation of αPD-L1 in the liver, followed by the spleen and lungs (Fig. 5c). Correspondingly, we found that MC38-EVs notably increased the uptake of αPD-L1 by blood monocytes and F4/80^+^ macrophages of the liver and spleen (Fig. 5d). MC38-EVs also increased the localization of αPD-L1 in F4/80^+^ macrophages in the liver and spleen (Fig. 5e and Supplementary Fig. 5d). These results suggest that TEVs alter αPD-L1 distribution in vivo. To directly verify that the binding of endogenous TEVs affects the in vivo distribution of αPD-L1, we transferred αPD-L1 into the MC38 *Rab27a*^*−/−*^ tumor-bearing mice. Compared with the MC38 tumor-bearing mice, the MC38 *Rab27a*^*−/−*^ tumor-bearing mice showed decreased liver distribution and increased tumor distribution (Fig. 5f). We observed a similar tendency in the MC38 *Coro1a*^*−/−*^ tumor-bearing mice (Supplementary Fig. 5e). Consistently, decreased αPD-L1 was observed in blood monocytes and liver and spleen macrophages of the MC38 *Rab27a*^*−/−*^ tumor-bearing mice (Fig. 5g, h and Supplementary Fig. 5f). Therefore, these results suggest that after binding TEVs, increased αPD-L1 is taken up by phagocytes, leading to accelerated degradation and decreased tumor delivery of αPD-L1.

Then, we determined whether the enhanced therapeutic effect of αPD-L1 can be achieved by targeting macrophages. We confirmed that pexidartinib (PLX3397), an inhibitor of colony-stimulating factor 1 receptor (CSF-1R), markedly reduced the numbers of peripheral monocytes and liver macrophages (Supplementary Fig. 5g). In the MC38 tumor-bearing mice, PLX3397 showed a significantly synergistic effect on αPD-L1 (Fig. 5i). In addition, decreasing αPD-L1-bound EVs-TT and increasing αPD-L1-bound tumor PD-L1 could be simultaneously observed in the PLX3397-treated mice (Fig. 5j, k), indicating the dissociation of αPD-L1 from TEVs. Then, we used clodronate liposomes (Clodrosomes) to specifically deplete macrophages and found that Clodrosomes also significantly improved the therapeutic effect of αPD-L1 (Supplementary Fig. 5h). Next, we used the MC38 *Rab27a*^*−/−*^ tumor-bearing mice to elucidate the role of TEVs in the PLX3397-mediated enhanced antitumor effect of αPD-L1. In these tumor-bearing mice, the synergistic effect of PLX3397 was completely abolished (Fig. 5l). More importantly, we found that depletion of macrophages by PLX3397 eliminated αPD-L1 therapy resistance in TRAMP-C2-bearing mice (Fig. 5m). These results demonstrate that targeting macrophages effectively prevents the clearance of TEV-bound αPD-L1, thus improving the utilization efficiency and therapy resistance of αPD-L1.

### TEVs inhibit the antitumor effect of αPD-L1 on human tumors

**Fig. 6:**
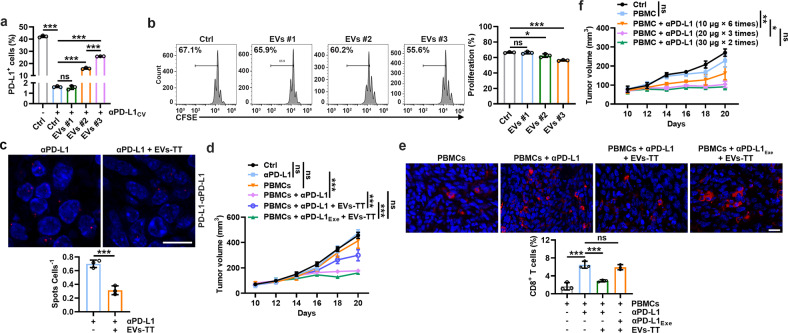
TEVs inhibit the antitumor effect of αPD-L1 on human tumors by consuming αPD-L1. **a** PC3 cells (1 × 10^5^) were coincubated with αPD-L1_CV_ and EVs from the sera of three lung tumor patients in 100 μl of medium for 30 min. Then, PD-L1 on the cells was detected by flow cytometry. **b** CFSE-labeled CD8^+^ T cells were stimulated with 2 μg ml^-1^ anti-CD3 and anti-CD28 for 24 h and then coincubated with 5 × 10^4^ PC3 cells and αPD-L1_CV_ with 10 μg of the indicated EVs in 200 μl of medium for 48 h. Then, the CFSE dilution was measured by flow cytometry. **c**–**f**, NOD-SCID mice with PC3 tumors were intratumorally injected with 1 × 10^6^ preactivated human peripheral blood mononuclear cells once when the tumor size reached 80–100 mm^3^. Two days later, the mice were intravenously injected with 10 μg of αPD-L1 or αPD-L1_Exe_ with or without 20 μg of EVs-TT (**c**–**e**), or the mice were intravenously injected with αPD-L1 according to the indicated strategies (**f**) every 2 days. The interaction of αPD-L1 and tumor PD-L1 was detected by PLA on Day 20 (**c**), the tumor sizes were monitored every other day (**d**, **f**), and CD8^+^ T cells in TTs were detected by immunofluorescence (**e**). Scale bar, 20 μm. Representative results from two independent experiments are shown (*n* = 3 except for *n* = 5 in **i**, **l**). *P < 0.05; ***P < 0.001; ns not significant (one-way ANOVA followed by Tukey’s test except for unpaired two-tailed Student’s *t test* in (**c**); mean and s.d.)

To extend our findings to humans, we isolated serum EVs from 3 lung tumor patients. EVs #1 were negative for PD-L1, while EVs #2 and #3 were positive for PD-L1 with higher PD-L1 levels on EVs #3 (Supplementary Fig. 6a). At αPD-L1_CV_, EVs #2 and #3 but not EV #1 increased αPD-L1-free PD-L1 on PC3 cells, and EV #3 had a stronger ability to dissociate αPD-L1 from tumor PD-L1, which was consistent with their ability to inhibit the αPD-L1-mediated rescue of CD8^+^ T-cell proliferation (Fig. 6a, b). In addition, we confirmed that EVs #2 and #3 (from 200 μl of serum) could bind approximately 14.70 ± 0.84 and 36.62 ± 1.19 ng (mean ± s.d.; *n* = 3) of αPD-L1. We also detected PD-L1 on the EVs-TT of another 3 lung cancer patients (Supplementary Fig. 6b) and found that each EVs-TT (from 1 mg TT) could bind approximately 6.04 ± 3.04, 16.55 ± 2.97 and 45.38 ± 4.48 ng (mean ± s.d.; *n* = 3) of αPD-L1. Then, we established a PC3 tumor model in nonobese diabetes/severe combined immune deficiency (NOD/SCID) mice, and in these tumor-carrying mice, EVs-TT #3 obviously decreased the binding of αPD-L1 and tumor PD-L1 (Fig. 6c). Treatment with αPD-L1 greatly inhibited tumor progression when PC3 tumor-carrying mice were simultaneously intratumorally injected with human peripheral blood mononuclear cells (PBMCs), which was significantly blunted by EVs-TT (Fig. 6d). However, EVs-TT did not affect the antitumor function of αPD-L1 when αPD-L1_Exe_ was used (Fig. 6d). In accordance with these results, EVs-TT reduced CD8^+^ T cells in TTs from αPD-L1- but not αPD-L1_Exe_-treated tumor mice (Fig. 6e). Furthermore, high-dose and low-frequency αPD-L1 treatment showed similarly improved antitumor effects in this tumor model (Fig. 6f). Thus, these results suggest that human TEVs impair the antitumor effect of αPD-L1 by consuming them.

### TEV PD-L1 causes different therapeutic outcomes for αPD-L1 and αPD-1

**Fig. 7:**
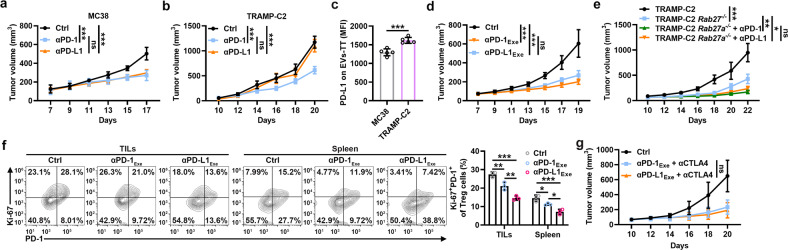
TEV PD-L1 causes different therapeutic outcomes for αPD-L1 and αPD-1. **a**–**c**, Mice with MC38 (**a**) or TRAMP-C2 (**b**) tumors were intravenously injected with 10 μg of αPD-1 or αPD-L1 every 2 days starting when the tumor size reached 80–100 mm^3^. The tumor size was monitored every other day (**a**, **b**). The PD-L1 levels on EVs-TT of these mice were detected by flow cytometry before αPD-1 or αPD-L1 treatment (MFI, mean fluorescence intensity) (**c**). **d**, **e** Mice with TRAMP-C2 (**d**) TRAMP-C2 *Rab27a*^*−/−*^ (**e**) tumors were intravenously injected with 30 μg (**d**) or 10 μg (**e**) of αPD-1 or αPD-L1 every 2 days starting when the tumor size reached 80–100 mm^3^. The tumor size was monitored every other day. **f** The frequency of Ki-67^+^PD-1^+^ Treg cells in TILs and spleens of mice in (**d**) was detected by flow cytometry on Day 19. **g** Mice with TRAMP-C2 tumors were intravenously injected with 20 μg of αCTLA-4 combined with 30 μg of αPD-1 or αPD-L1 every 2 days starting when the tumor size reached 80–100 mm^3^. The tumor size was monitored every other day. Representative results from two independent experiments are shown (*n* = 5 except for *n* = 3 in **c**, **f**). *P < 0.05; **P < 0.01; ***P < 0.001; ns not significant (one-way ANOVA followed by Tukey’s test except for unpaired two-tailed Student’s *t* test in (**c**); mean and s.d.)

As mentioned above, TEVs specifically attenuate the ability of αPD-L1 but not αPD-1 to rescue CD8^+^ T-cell proliferation, so TEVs probably lead to the different antitumor effects of αPD-L1 and αPD-1. In αPD-L1-resistant TRAMP-C2 but not αPD-L1-sensitive MC38 tumor-bearing mice [12], we observed that αPD-1 had better therapeutic effects than αPD-L1 (Fig. 7a, b). Correspondingly, the PD-L1 levels on EVs-TT of the MC38 and TRAMP-C2 tumor-bearing mice had an opposite trend before treatment (Fig. 7c). To elucidate the role of TEVs in this process, we first confirmed that supplementation with TEVs did not affect the antitumor effect of αPD-1 (Supplementary Fig. 7a, b). Consistent with these results, an increased αPD-1 dose did not improve the therapeutic effect on the TRAMP-C2-bearing mice (Supplementary Fig. 7c). However, αPD-L1_Exe_ and αPD-1_Exe_ showed comparable effects against TRAMP-C2 (Fig. 7d). Furthermore, a comparable antitumor effect was observed in the TRAMP-C2 *Rab27a*^*−/−*^ tumor-bearing mice treated with low and high doses of αPD-L1 and αPD-1 (Fig. 7e and Supplementary Fig. 7d). These results suggest that TEVs specifically blunt the antitumor effect of αPD-L1.

αPD-1 treatment blocks PD-1 signaling in all subsets of T cells, which amplifies PD-1^+^ regulatory T (Treg) cells, thereby leading to hyperprogression of cancer [22]. However, αPD-L1 treatment blocked PD-L1 but not PD-L2, which may restrain the amplification of PD-1^+^ Treg cells. We indeed found in the TRAMP-C2 tumor-bearing mice that αPD-1_Exe_ treatment induced more Ki-67^+^PD-1^+^ Treg cells than αPD-L1_Exe_ treatment (Fig. 7f). In addition, PD-L2 inhibited the proliferation of PD-1^+^ Treg cells in vitro (Supplementary Fig. 7e). From this perspective, the antitumor effect of αPD-L1_Exe_ should be better than that of αPD-1_Exe_. However, we did not observe this result (Fig. 7d). PD-L1 can form heterodimers with CD80 and disrupt the interaction of CD80 and CTLA-4, causing the inhibition of CTLA-4 signaling [23]. Therefore, αPD-L1 but not αPD-1 treatment probably enhances the activation of CTLA-4 signaling, which specifically blunts the antitumor effect of αPD-L1. However, αPD-L1_Exe_ and αCTLA-4 combination therapy showed similar therapeutic effects to αPD-1_Exe_ and αCTLA-4 combination therapy in the TRAMP-C2 tumor-bearing mice (Fig. 7g). In summary, these results indicate that when αPD-L1 is sufficient, αPD-L1 and αPD-1 have comparable antitumor effects.

## Discussion

Although TEVs may mediate αPD-L1 therapy resistance [12], their definite role in this process has yet to be explored. In addition, how TEVs mediate αPD-L1 therapy resistance is unknown. In this study, we found that TEVs could decoy αPD-L1 in large quantities via PD-L1. EVs from MC38 TTs bound increased αPD-L1 with tumor progression, and EVs from MC38 TTs of 4-week tumor-bearing mice could bind approximately 2.51 μg of αPD-L1, almost 25.10% of the therapeutic dose (10 μg). Furthermore, in some patients, EVs from 1 mg TTs of tumor patients bound approximately 45.38 ng of αPD-L1. The therapeutic dose of αPD-L1 in the clinic is 1200 mg. It has been reported that the tumor weight of patients with malignant pleural mesothelioma can reach 983 g [24]. Of the 44 patients with adrenocortical carcinoma, 9 had tumors weighing more than 1000 g [25]. Therefore, in some tumor patients, total EVs-TT can bind more than 45.38 mg of αPD-L1, which is >3.78% of the therapeutic dose of αPD-L1. Furthermore, TEVs are continuously secreted and simultaneously present in the circulation and various organs. Therefore, the actual amount of TEVs in the body is much higher. In addition, not all the injected αPD-L1 can permeabilize into tumors to be effectively utilized. Due to the small volume and large specific surface area of EVs, EV PD-L1 can easily enter deep tissues [26]. Thus, TEVs probably more efficiently decoy tumor-permeabilized αPD-L1. Collectively, TEV decoy-mediated consumption of αPD-L1 probably leads to insufficient αPD-L1 for therapy in patients with high levels of TEV PD-L1. As expected, a notably enhanced therapeutic effect was observed in the MC38 tumor-bearing mice when the dose of αPD-L1 was increased. More importantly, a high αPD-L1 dose also reversed αPD-L1 therapy resistance in TRAMP-C2 tumors. Thus, our results suggest that consumption of a large amount of αPD-L1 by TEVs leads to resistance to αPD-L1 therapy.

In addition to TEVs, PD-L1^+^ EVs can also be produced by other types of cells. TEVs upregulate PD-L1 expression in tumor-associated macrophages [27], while plasma membrane PD-L1 of parent cells may be the major source of EV PD-L1 [26]. Therefore, tumor-associated macrophages with upregulated membrane PD-L1 probably secrete EVs with high levels of PD-L1, which may also contribute to the decoy and consumption of αPD-L1. Moreover, T-cell-derived EVs carried levels of PD-L1 similar to those of tumor cells in head and neck squamous cell carcinoma [28]. Therefore, the parent cells of PD-L1^+^ EVs are diverse in vivo. Although TEV PD-L1 seems to be a more accurate predictor for immunotherapy [26], PD-L1 on other EVs should also have the ability to decoy αPD-L1. In addition to exosome-like EVs, our results showed that MVs from tumor cells also contained membrane-associated PD-L1 and could compete with tumor cells to bind αPD-L1. All these factors indicate that the pool of EVs mediating αPD-L1 consumption is much larger than previously thought.

To prevent the side effects of increasing the therapeutic dose as much as possible, we tried to develop a better treatment strategy without changing the total therapeutic dose. We found that at the same total therapeutic dose, high-dose and low-frequency treatment with αPD-L1 effectively overcame αPD-L1 therapy resistance in TRAMP-C2 tumors. We supposed that a sufficient dose of αPD-L1 therapy each time could induce antitumor immunity and establish antitumor immune memory more effectively. Antitumor immune memory is long-lasting and can prevent tumor recurrence. Therefore, even if the total therapeutic frequency is reduced, a better antitumor effect is achieved. We detected more memory T cells in the TRAMP-C2 tumor-bearing mice treated with high-dose and low-frequency αPD-L1. Therefore, we developed an effective strategy to overcome αPD-L1 therapy resistance.

Macrophages are the dominant effector cells mediating EV phagocytosis [21, 29]. Macrophages were also reported to capture αPD-1 from the T-cell surface via the Fcγ receptor [30]. We found that TEV-bound αPD-L1 was cleared by macrophages more quickly than free αPD-L1. Depletion of macrophages by PLX3397 led to the dissociation of αPD-L1 from TEVs and increased the blockade of PD-L1 on tumor cells, thereby synergizing with αPD-L1 and abolishing αPD-L1 therapy resistance. PLX3397 was recently approved by the Food and Drug Administration to treat tenosynovial giant cell tumors [31]. Therefore, combination with PLX3397 is a promising strategy to overcome αPD-L1 therapy resistance mediated by TEVs. PLX3397 also improves the antitumor effect of αPD-1, but in contrast to this study, which showed that PLX3397 promoted CD8^+^ T-cell infiltration into tumors, we propose that PLX3397 probably enhances the antitumor effect of αPD-L1 by increasing the utilization of αPD-L1.

Consistent with previous studies, we detected PD-L1 on Circ-EVs of tumor patients [11, 32], but the amount of αPD-L1 bound by Circ-EVs was very low. The total Circ-EVs of tumor patients bound less than 1 mg of αPD-L1 (based on an adult with 4–5 l blood), which is almost negligible. However, we found that the PD-L1 levels of Circ-EVs were positively correlated with the PD-L1 levels of EVs-TT in tumor-bearing mice. Therefore, the PD-L1 levels of Circ-EVs can reflect those of EVs-TT and predict the outcome of αPD-L1 therapy, and the αPD-L1-therapy regimen may also need to be rationally adjusted according to the PD-L1 levels of Circ-EVs. In addition, our results showed that TEV PD-L1 did not affect the antitumor effect of αPD-1. However, Circ-EVs of tumor patients have been demonstrated to predict the response to αPD-1 therapy [32]. High TEV PD-L1 will likely cause T-cell exhaustion, thereby blunting the αPD-1 therapeutic effect, which makes circulating EV PD-L1 an effective predictor of the response to αPD-1 therapy.

αPD-1 blocks the activation of PD-1 signaling induced by both PD-L1 and PD-L2, while αPD-L1 prevents only the PD-L1-mediated activation of PD-1 signaling. According to our results, the preservation of PD-L2 function probably prevents the amplification of PD-1^+^ Treg cells. When the consumption of αPD-L1 by TEV PD-L1 is eliminated by using excess αPD-1, they can achieve comparable therapeutic effects to αPD-1. Simultaneously, αPD-L1 will induce fewer PD-1^+^ Treg cells, thus reducing cancer hyperprogression. PD-L1 can form heterodimers with CD80 on antigen presenting cells and disrupt the interaction of CD80 and CTLA-4, thereby attenuating CTLA-4 signaling [23]. Furthermore, PD-L1 interacts specifically with CD80 on T cells to inhibit T-cell responses [33], which can be blocked only by αPD-L1. However, in combination with αCTLA-4, we did not observe a better therapeutic effect of αPD-L1 than that of αPD-1. This result suggests that the functions of αPD-L1 are far more complex than the current model. However, our results also indicate that αPD-1 and αPD-L1 are not simply alternatives to each other.

## Materials and methods

### Human samples

TTs from lung cancer patients and blood from healthy volunteers were obtained from the Second Affiliated Hospital, Zhejiang University School of Medicine and approved by the Ethics Committee. All the patients and healthy volunteers were informed of the use of their samples, and signed consent forms were obtained.

### Mice

C57BL/6 J and NOD/SCID female mice aged 6-8 weeks were purchased from Joint Ventures Sipper BK Experimental Animal Co. (Shanghai, China). *Foxp3*^*GFP*^ knock-in C57BL/6 mice were generously provided by Prof. Zhexiong Lian (South China University of Technology, Guangzhou, Guangdong, China). The mice were housed in a specific pathogen-free facility, and the experimental protocols were approved by the Animal Care and Use Committee of the First Affiliated Hospital of Zhejiang University.

### Cell lines and cell culture

PC3 cells, MC38 cells and TRAMP-C2 cells were purchased from the Chinese Academy of Sciences Institute (Shanghai, China). PC3, MC38 and TRAMP-C2 cells were cultured in DMEM with 10% exosome-depleted fetal bovine serum (FBS) (Thermo Fisher Scientific, Waltham, CA, USA) and 1% penicillin/streptomycin (Keyi, Hangzhou, Zhejiang, China). PMs were collected 3 days after the intraperitoneal injection of C57BL/6 J mice with thioglycolate (Millipore, Billerica, MA, USA). PMs were cultured in RPMI-1640 with 10% FBS and 1% penicillin/streptomycin. All cells were cultured at 37 °C with 5% CO_2_.

### Separation of EVs

PC3, MC38 and TRAMP-C2 cells were plated at a density of 3 million cells per 15-cm plate (Corning 430599) and cultured for 48 h, and the media from 10 plates were collected. For PC3-EV, MC38-EV and B16-EV separation, cell culture supernatants were centrifuged at 300 × *g* for 10 min, 2000 × *g* for 20 min and 10,000 × *g* for 30 min at 4 °C. Then, the MV pellets were resuspended in sterile PBS, and the supernatants were passed through 0.22 μm syringe filters (Millipore, Darmstadt, Germany) and collected in 35 ml ultracentrifuge tubes (Beckman Coulter, Brea, CA, USA). The EVs were concentrated using ultracentrifugation with a SW32Ti rotor (L-90K with SW32Ti rotor, Beckman Coulter) at 100,000 × *g* for 70 min at 4 °C. Subsequently, the EV pellets were resuspended in sterile PBS. The protein contents of the EVs were quantified by using a BCA protein assay kit in the absence of detergent (Thermo Fisher Scientific).

### EM

A total of 5 μg of PC3-EVs or MC38-EVs was diluted in PBS and placed on 200-mesh carbon-coated copper grids at room temperature (RT) for 2 min. The excess suspension was removed using filter paper. Then, the PC3-EVs or MC38-EVs were negatively stained with uranyl acetate at RT for 5 min, washed twice with PBS, dried and examined under an FEI Tecnai T10 EM (FEI, Hillsboro, OR, USA) operating at 100 kV.

### Western blotting

Equal amounts of cell or tissue lysate or EV proteins were resuspended in 5 × SDS loading buffer, incubated at 100 °C for 5 min, and centrifuged at 12,000 × *g* for 10 min. Samples were separated by 10% SDS-polyacrylamide gel electrophoresis and transferred to PVDF membranes (Millipore), which were blocked with 5% milk for 1.5 h, incubated with the corresponding primary antibodies at 4 °C overnight, and then incubated with secondary antibodies at RT for 2 h. An Enhanced Chemiluminescence Kit (MultiSciences, Hangzhou, Zhejiang, China) was used to detect the bands. The antibodies used and the corresponding dilutions are listed in Supplementary Table 1.

### Nanoparticle tracking analysis

The number and size distribution of EVs were analyzed using a NanoSight NS300 (Malvern, Malvern, Worcestershire, UK). EVs were resuspended in PBS for analysis. For recordings, samples were pumped automatically into a chamber at a constant flow rate using the Malvern NanoSight syringe pump system. The camera level was adjusted to 14, and three 30’ captures per sample were recorded. For analysis of the recordings, the detection threshold was set to 5, and the NTA3.3 Suite Software was used for analysis.

### Flow cytometry analysis

Cells or EVs incubated with 4-μm aldehyde sulfate beads (Thermo Fisher Scientific) were washed in PBS with 1% BSA, collected by centrifugation at 400 × *g* or 3500 × *g* for 5 min at 4 °C, and then incubated with the corresponding fluorescence-conjugated primary antibodies in 100 μl of PBS at predetermined saturating concentrations for 20 min at RT. After two washes in PBS, the cells or beads were analyzed on an ACEA NovoCyte flow cytometer (ACEA Biosciences, San Diego, CA, USA). For intracellular staining, cells were stimulated with PMA (50 ng ml^−1^, Sigma–Aldrich, St. Louis, MO, USA), ionomycin (1 μg ml^−1^, Sigma–Aldrich), and brefeldin A solution (eBioscience, San Diego, CA, USA) at 37 °C for 4 h and then subjected to intracellular staining. The data were analyzed using FlowJo software (Tree Star, Ashland, OR, USA), and the antibodies used and the corresponding dilutions are listed in Supplementary Table 1.

### CRISPR–Cas9-mediated depletion of Rab27a or PD-L1

For depletion of Rab27a or PD-L1 in MC38 cells, the guide RNA plasmid (gRNA; sequences are listed in Supplementary Table 2) was cloned into pLentiCRISPR V2 (Miaolingbio, Wuhan, Hubei, China). After 48 h of transfection of the plasmids into MC38 cells, the cells were selected with 2 μg ml^−1^ puromycin. Live cells were sorted using a Beckman Coulter DxFLEX flow cytometer (Beckman Coulter). After sorting, single cells were cultured in 96-well plates. The Rab27a or PD-L1 knockout efficiency was confirmed by western blotting or flow cytometry. Selected MC38 cells with unchanged Rab27a or PD-L1 expression were used as controls.

### In vitro T-cell proliferation assays

Mouse CD8^+^ T cells were isolated from splenocytes and peripheral lymph nodes with a Mouse CD8^+^ T-Cell Isolation Kit (StemCell, Vancouver, BC, Canada). Human CD8^+^ T cells were isolated from PBMCs of healthy donors with a Human CD8^+^ T-Cell Enrichment Kit (StemCell). A total of 1 × 10^6^ CD8^+^ T cells were labeled with CFSE (Thermo Fisher Scientific) at 5 μM. The cells were then incubated at 37 °C for 5 min, and the reaction was stopped by adding an equal volume of RPMI-1640 with 10% FBS. Unstimulated CFSE-labeled cells served as a nondividing control. Both mouse and human CD8^+^ T cells (1 × 10^6 ^ml^−1^) were stimulated with αCD3 and αCD28 (2 μg ml^−1^, Bio X Cell, West Lebanon, NH, USA) for 24 h and then incubated with MC38 and PC3 cells alone (2.5 × 10^5 ^ml^−1^) or MC38 and PC3 cells plus the corresponding TEVs with or without αPD-L1 (BioLegend, San Diego, CA, USA) for 48 h.

PD-1^+^ Treg cells were isolated from splenocytes and peripheral lymph nodes in *Foxp3*^*GFP*^ transgenic mice and sorted by a Beckman Coulter DxFLEX flow cytometer (Beckman Coulter). A total of 1 × 10^6^ PD-1^+^ Treg cells were labeled with 1 μM CellTrace^TM^ Far Red (Thermo Fisher Scientific). Then, the reaction was stopped by adding an equal volume of RPMI-1640 with 10% FBS, and the cells (3 × 10^5^ ml^−1^) were stimulated with complete RPMI 1640 medium containing 1 ng ml^−1^ PMA, 200 ng ml^−1^ ionomycin (MedChemExpress, Monmouth Junction, NJ, USA), and 4000 U ml^−1^ murine IL-2 (R&D, Minneapolis, MN, USA) in the presence of 5 μg ml^−1^ recombinant mouse PD-L2 (BioLegend) for 72 h.

### ELISA

For determination of the αPD-L1-binding ability of EVs, 96-well ELISA plates were coated with αCD63, αCD81 and αCD9 at 4 °C overnight (0.1 μg per well, BioLegend). Free binding sites were blocked with 100 μl of blocking buffer for 1 h at RT. Then, serum samples or EVs-TT (50 μl per sample) were added to duplicate wells, followed by incubation overnight at 4 °C. The plates were washed, and biotinylated αPD-L1 (Thermo Fisher Scientific) or biotin αRat IgG (BioLegend) was added to each well and incubated for 1 h at RT. Then, streptavidin-HRP (BioLegend) diluted in 100 μl of PBS was added and incubated for 1 h at RT. The reaction was developed with TMB and blocked with 2 M H_2_SO_4_, followed by measurement of the absorbance at 450 nm. The concentration of αPD-L1 on the surface of EVs was calculated based on the linear range of the ELISA data. Serial dilutions of biotinylated αPD-L1 (Thermo Fisher Scientific) were used to generate a standard curve. The results of the standard curve demonstrated that the established ELISA exhibited a reliable linear detection range from 3 to 800 ng ml^−1^.

### Animal studies

For construction of subcutaneous tumor models, MC38, MC38 *Rab27a*^*−/−*^, MC38 *Coro1a*^*−/−*^, TRAMP-C2 and TRAMP-C2 *Rab27a*^*−/−*^ cells (2 × 10^6^) were resuspended in 200 μl of PBS and subcutaneously implanted into the right flank of C57BL/6 female mice on Day 0. PC3 cancer cells (5 × 10^6^) were injected subcutaneously into NOD-SCID mice on Day 0. When tumors reached an average of 100–200 mm^3^, as calculated with the formula volume = (width^2^ × length) 2^−1^, the mice were randomized into different treatment groups. For analysis of the treatment difference between αPDL1 and αPD-1, 10 μg of αPD-1 (BioLegend) or αPD-L1 was injected intravenously into mice every 2 days. For determination of the effect on TEVs binding αPD-L1 in vivo, the mice with TRAMP-C2, TRAMP-C2 *Rab27a*^*−/−*^, MC38, MC38 *Rab27a*^*−/−*^ or MC38 *Coro1a*^*−/−*^ tumors were intravenously injected with 10 or 30 μg of αPD-L1 every 2 days. For determination of whether TEV-bound αPD-L1 is eliminated by macrophages, the mice with MC38 or MC38 *Rab27a*^*−/−*^ tumors were intravenously injected with 10 μg of αPD-L1 with or without intraperitoneal injection of 20 μg of PLX3397 (MedChemExpress, Monmouth Junction, NJ, USA) or with or without intravenous injection of 50 μl Clodrosomes (Clodrosome, Brentwood, TN, USA) every 2 days when the tumor size reached 100–200 mm^3^. For the humanized tumor model, NOD-SCID mice were intratumorally injected with preactivated human PBMCs (1 × 10^6^) when the tumor size reached 80–100 mm^3^. Two days later, the mice were intravenously injected with αPD-L1, with or without 20 μg PC3-EVs. In some experiments, the mice with TRAMP-C2 tumors were intravenously injected with 20 μg of αCTLA-4 (BioLegend) and 30 μg of αPD-L1 or αPD-1 every 2 days when the tumor size reached 80–100 mm^3^. At the experimental end point, livers, spleens and tumors were excised for subsequent histologic analysis or processed immediately for flow cytometry analyses, and serum was collected for ELISAs.

### PLA

Murine tumor tissue sections were routinely deparaffinized and rehydrated, followed by antigen retrieval using 10 mM sodium citrate buffer (pH 6.0). After the samples were blocked with 1× blocking solution at 37 °C for 1 h, they were incubated with mouse αRat IgG2a (BioLegend) and rabbit αPD-L1 (ABclonal, Wuhan, Hebei, China) overnight at 4 °C. Then, PLA was performed with Duolink In situ reagents (Sigma–Aldrich, St. Louis, MO, USA) according to the manufacturer’s instructions. Then, the samples were imaged using Olympus FluoView version 1.4a software (Olympus, Tokyo, Japan). Images of cells and sections were acquired, and positively stained areas were analyzed by ImageJ software (NIH, Bethesda, MD, USA).

### In vivo images

αPD-L1 was labeled with Protein Labeling Kits (Thermo Fisher Scientific) according to the manufacturer’s instructions. Then, 10 μg of labeled αPD-L1 or 10 μg of labeled αPD-L1 and 20 μg of TEV mixture were intravenously injected into mice. 12 h later, the mice were sacrificed, and the brain, heart, lungs, liver, spleen, kidneys, gut and tumor were collected. The labeled αPD-L1 was imaged by an IVIS (PerkinElmer, Waltham, MA, USA). The background and autofluorescence were defined according to the supernatant negative controls and subtracted from the images using the Image-Math function. In addition, the exposure conditions (time, aperture, stage position, and binning) were identical for all measurements within each experiment. Total measurements were obtained under the same conditions for all experimental groups.

### Immunofluorescence

The murine PMs were cultured overnight on glass coverslips and then treated with lysosome inhibitors for 24 h. Then, the cells were coincubated with labeled αPD-L1 or TEV-bound αPD-L1 for another 2 h. After being washed three times with PBS, the cells were fixed with precooled methyl alcohol for 10 min at −20 °C and then permeabilized with 0.1% Triton X-100 for 10 min at RT. After the cells were blocked with 5% BSA and 3% goat serum in PBS, they were incubated with LAMP1 antibodies (Abcam, Cambridge, UK) overnight at 4 °C in blocking buffer. The following day, after three washes in PBS, the cells were incubated with DyLight 488-labeled secondary antibodies (Multi Sciences Biotech, Hangzhou, China) for 30 min at RT and washed in PBS. Finally, nuclei were stained with DAPI (Thermo Fisher Scientific). Liver and spleen tissues were embedded in Tissue-Tek™ CRYO-O.C.T. (Thermo Fisher Scientific) and processed to obtain 5 μm sections. Then, the tissue sections were stained with mouse F4/80 antibodies (Abcam) at 4 °C overnight followed by staining with DyLight 488-labeled secondary antibodies (Multi Sciences Biotech) for 1 h at 4 °C. The nuclei were stained with DAPI (Thermo Fisher Scientific) for 20 min at RT. The stained sections were imaged using an Olympus IX83-FV3000 confocal microscope (Olympus). Images were analyzed with ImageJ software (NIH).

### Statistical analysis

All statistical analyses were performed using GraphPad Prism 8.0 (GraphPad Software, Inc., San Diego, CA, USA). All data are expressed as the mean ± s.d. An unpaired two-tailed Student’s *t* test was used to compare the differences between two groups, and one-way ANOVA followed by Tukey’s test was used to compare the differences among multiple groups. The Spearman rank-order correlation test was used for correlation analysis. A difference was considered significant if the *P* value was < 0.05.

## Supplementary information

Supplementary Information

## Data Availability

All data needed to evaluate the conclusions in the paper are presented in the paper. Materials described in the study are either commercially available or available upon request from the corresponding author.
