# Supplementary material for: Tumor extracellular vesicles mediate anti-PD-L1 therapy resistance by decoying anti-PD-L1
Source: Cell Mol Immunol. 2022 Oct 11;19(11):1290–301. doi: 10.1038/s41423-022-00926-6 (PMC9622748; doi:10.1038/s41423-022-00926-6)
Supplement: Supplementary file 1 — Supplementary Information [file 41423_2022_926_MOESM1_ESM.pdf]

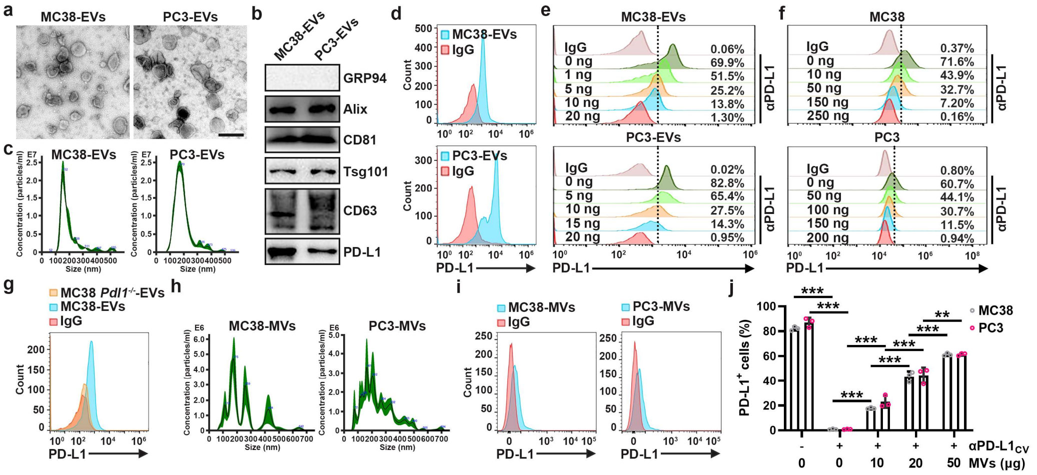

Supplementary Fig. 1 TEV PD-L1 competes with PD-L1 on tumor cells to bind αPD-L1. **a**, The morphology of EVs was detected by electron microscopy (EM). Scale bar, 100 nm. **b**, The indicated proteins in EVs were detected by western blotting. **c**, The size distribution of EVs was analyzed by nanoparticle tracking analysis. **d**, PD-L1 on MC38-EVs and PC3-EVs was detected by flow cytometry. **e**, **f**, One microgram of MC38-EVs and PC3-EVs (**e**) or 1×10^5 MC38 and PC3 cells (**f**) were coincubated with the indicated doses of αPD-L1 in 100 μl of medium for 30 min. Then, PD-L1 on EVs (**e**) or cells (**f**) was detected by flow cytometry, and representative histograms are shown. The αPD-L1 for coincubation and detection recognizes the same epitope in PD-L1. **g**, PD-L1 on MC38 *Pdl1*$^{-/-}$-EVs was detected by flow cytometry. **h**, The size distribution of MC38-MVs and PC3-MVs was analyzed by nanoparticle tracking analysis. **i**, PD-L1 on MC38-MVs and PC3-MVs was detected by flow cytometry. **j**, A total of 1×10^5 MC38 and PC3 cells were coincubated with αPD-L1$_{CV}$ with or without 50 μg of MC38-MVs or PC3-MVs in 100 μl of medium for 30 min. Then, PD-L1 on the cells was detected by flow cytometry. Representative results from three independent experiments are shown ($n = 3$). $^{**}P < 0.01$; $^{***}P < 0.001$ (unpaired two-tailed Student's *t*-test; mean and s.d.).

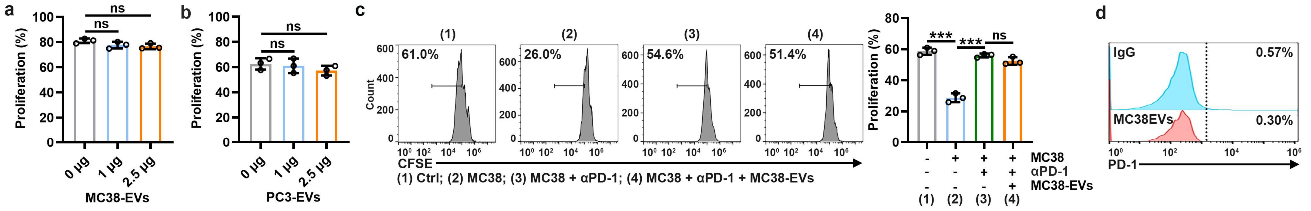

Supplementary Fig. 2 TEVs impair αPD-L1-induced $CD8^+$ T-cell proliferation by consuming αPD-L1. **a**, **b**, CFSE-labeled $CD8^+$ T cells were stimulated with 2 μg ml^{-1} anti-CD3 and anti-CD28 for 24 h and then treated with the indicated doses of MC38-EVs (a) or PC3-EVs (b) in 200 μl of medium for 48 h. Then, the proliferation of $CD8^+$ T cells was assessed according to CFSE dilution by flow cytometry. **c**, CFSE-labeled $CD8^+$ T cells were stimulated with 2 μg ml^{-1} anti-CD3 and anti-CD28 for 24 h and then coincubated with 5×10^4 MC38 and αPD-1 with or without 2.5 μg of MC38-EVs in 200 μl of medium for 48 h. Then, the CFSE dilution was measured by flow cytometry. d, PD-1 on MC38-EVs was detected by flow cytometry. Representative results from three independent experiments are shown (n = 3). ***P < 0.001; ns, not significant (one-way ANOVA followed by Tukey's test; mean and s.d.).

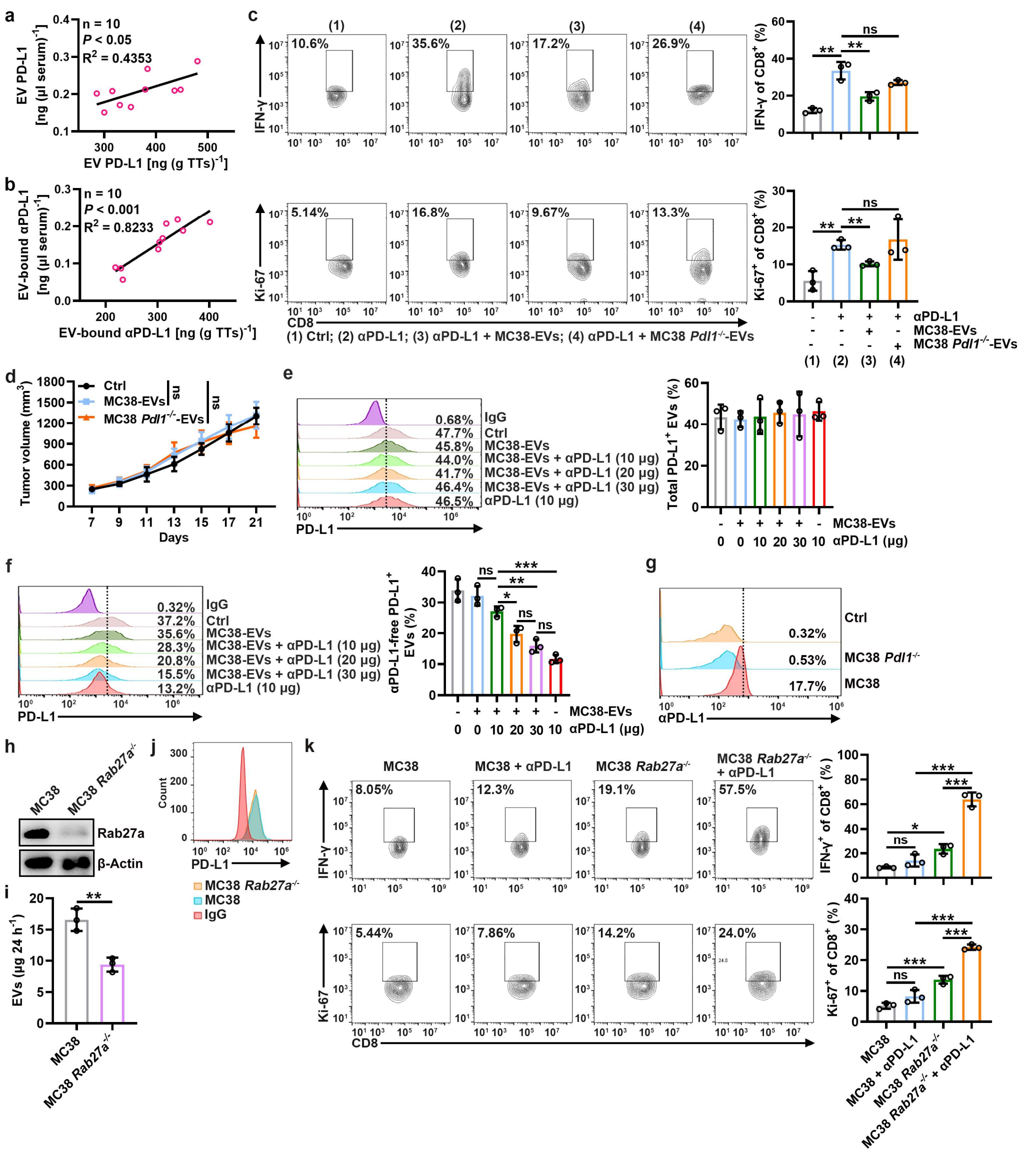

Supplementary Fig. 3 TEV-mediated αPD-L1 consumption blunts the antitumor effect of αPD-L1. **a**, **b**, PD-L1 levels on Circ-EVs and EVs-TT or αPD-L1 amount bound by Circ-EVs and EVs-TT were detected by ELISA. The correlation of PD-L1 levels on Circ-EVs and EVs-TT (**a**) and the amounts of αPD-L1 bound by Circ-EVs and EVs-TT (**b**) were analyzed. **c**, IFN-γ^+CD8$^+$ and Ki-67$^+$CD8$^+$ T cells in TTs of mice in Fig. 3c were analyzed by flow cytometry. **d**, Mice with MC38 tumors were intravenously injected with 20 μg of MC38-EVs or MC38 *Pdl1*$^{-/-}$-EVs every 2 days starting when the tumor size reached 100-200 mm^3. Tumor sizes were monitored every other day. **e-g**, Total PD-L1 was detected by αPD-L1 recognizing distinct epitope in PD-L1 to therapeutic αPD-L1 (**e**), αPD-L1-free PD-L1 was detected by

αPD-L1 recognizing the same epitope in PD-L1 to therapeutic αPD-L1 (**f**) or αPD-L1 (**g**) on EVs from TTs of mice in Fig. 3d (**e**, **f**) or mice with MC38 tumors or MC38 *Pdl1*$^{-/-}$ tumors (**g**) were analyzed by flow cytometry. **h-j**, Rab27a in (**h**), EV release from (**i**) or PD-L1 on (**j**) 1×10^7 MC38 and MC38 *Rab27a*$^{-/-}$ cells was detected by western blotting (**h**), quantified by BCA assay (**i**) or analyzed by flow cytometry (**j**). **k**, IFN-γ^+CD8$^+$ and Ki-67$^+$CD8$^+$ T cells in the TTs of mice in Fig. 3h were analyzed by flow cytometry. Representative results from two independent experiments are shown (n = 3 in **c**, **e**, **f**, **i**, **k;** n = 5 in **d**). *P < 0.05; **P < 0.01; ***P < 0.001; ns, not significant (Spearman rank-order correlation test in **a**, **b**; one-way ANOVA followed by Tukey's test in **c**, **d**, **f**, **k**; unpaired two-tailed Student's t-test in **i**; mean and s.d.).

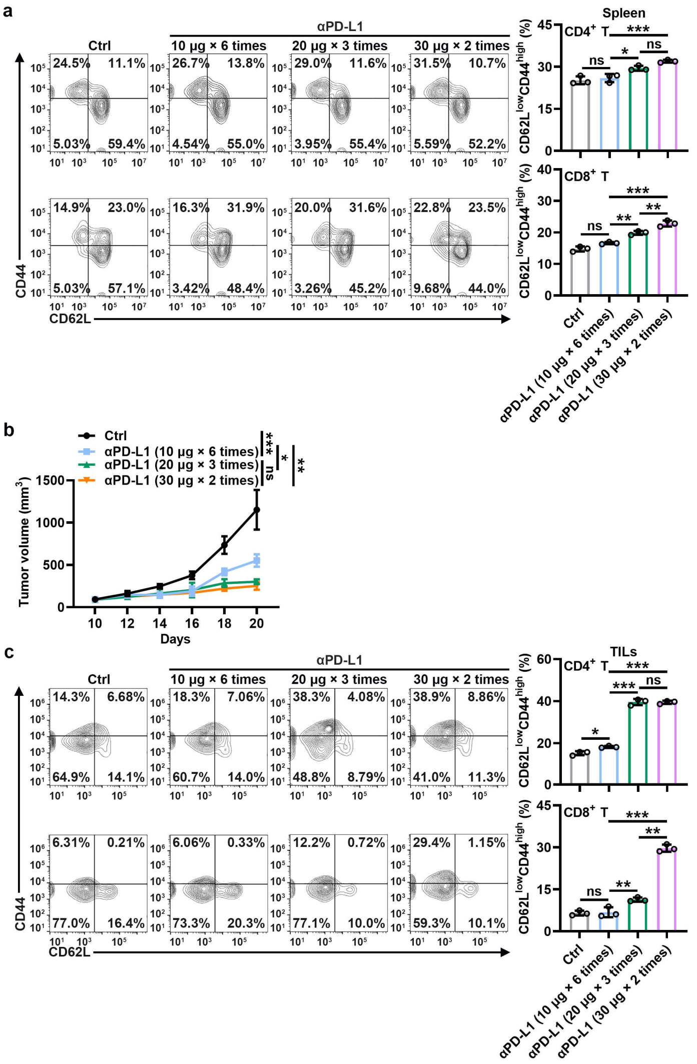

Supplementary Fig. 4 High-dose and low-frequency treatment reverses αPD-L1 therapy resistance. **a-c**, Mice with TRAMP-C2 (**a**) or MC38 (**b**, **c**) tumors were intravenously injected with αPD-L1 according to the indicated strategies every 2 days starting when the tumor size reached 100-200 mm^3. $CD62L^{low}CD44^{high}$ memory T cells in the spleen (**a**) and TILs (**c**) were analyzed by flow cytometry on Days 19 (**a**) and 20 (**c**). Tumor sizes were monitored every other day (**b**). Representative results from two independent experiments are shown (n = 3 in **a**, **c**; n = 5 in **b**). *P < 0.05; **P < 0.01; ***P < 0.001; ns, not significant (one-way ANOVA followed by Tukey's test; mean and s.d.).

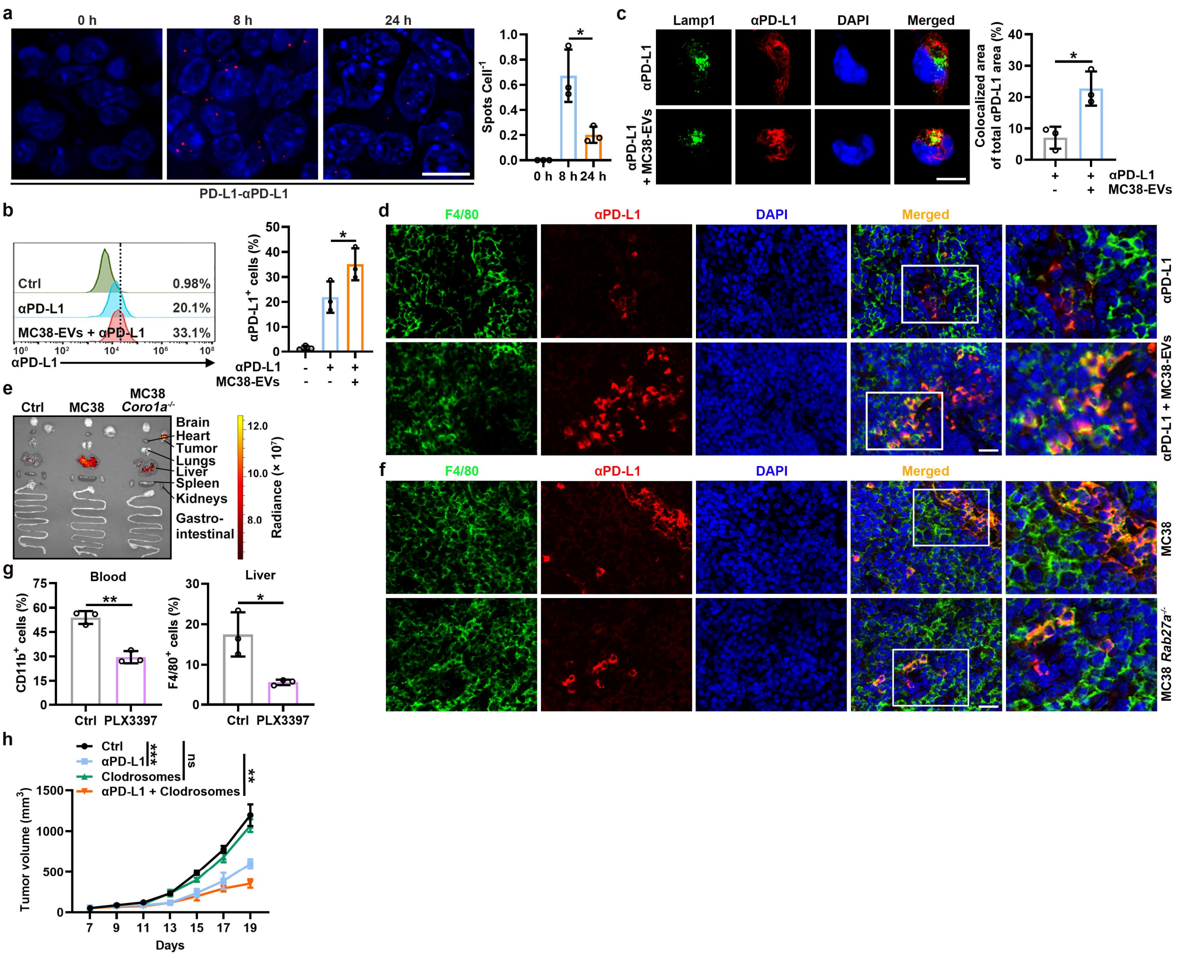

Supplementary Fig. 5 TEV-bound αPD-L1 is eliminated by macrophages. **a**, **b**, Mice with MC38 tumors were intravenously injected with 10 μg of αPD-L1 for the indicated time. Then, the interaction of αPD-L1 and tumor PD-L1 was detected by PLA. Scale bar, 10 μm. **b**, **c**, PMs were treated with 2 μg of αPD-L1 without or with 5 μg of MC38-EVs for 2 h. The phagocytosis of αPD-L1 was detected by flow cytometry (**b**), and the colocalization of αPD-L1 and lysosomes was detected by immunofluorescence. Scale bar, 10 μm (**c**). **d-f**, Mice without tumors (**d**), with MC38 or MC38 *Coro1a*$^{-/-}$ tumors (**e**), or with MC38 or MC38 *Rab27a*$^{-/-}$ tumors (**f**) were intravenously injected with 10 μg of Alexa Fluor 680-labeled αPD-L1 with (**d**) or without (**e**, **f**) 20 μg of MC38-EVs. The αPD-L1 in spleen macrophages was detected by immunofluorescence (scale bar, 20 μm) at 24 h (**d**) or 21 days after tumor cell injection (**f**). The distribution of αPD-L1 was detected by IVIS after 24 h (**e**). **g**, Mice were intraperitoneally injected with 20 μg of PLX3397 3 times at 2 day intervals, and monocytes in the blood and macrophages in the liver

were analyzed by flow cytometry 24 h after the last injection. **h**, Mice with MC38 tumors were intravenously injected with 10 μg of αPD-L1 with or without intraperitoneal injection of 50 μl of Clodrosomes every 2 days starting when the tumor size reached 100-200 mm^3. Tumor sizes were monitored every other day. Representative results from two independent experiments are shown ($n = 3$ except for n = 5 in **h**). *$P < 0.05$; **$P < 0.01$; ***$P < 0.001$ (unpaired two-tailed Student's *t*-test; mean and s.d.).

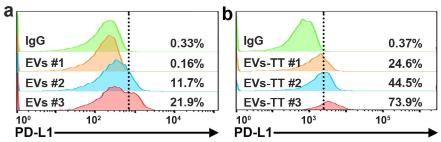

Supplementary Fig. 6 PD-L1 is presented on EVs from the sera and TTs of tumor patients. **a**, **b**, PD-L1 on EVs from sera (**a**) and TTs (**b**) from lung cancer patients was detected by flow cytometry. Representative results from three independent experiments are shown.

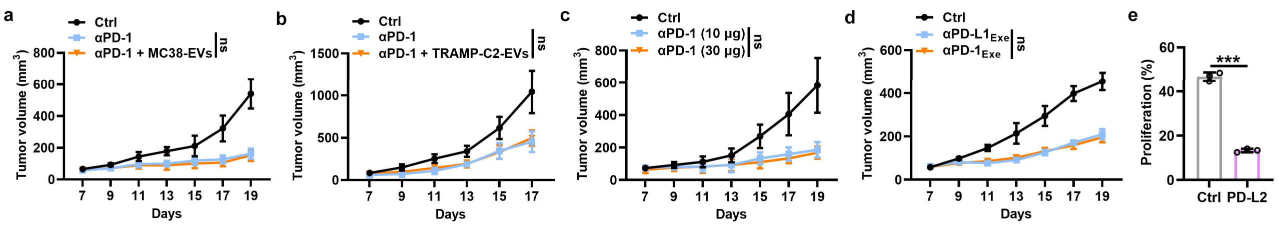

Supplementary Fig. 7 TEV PD-L1 causes different therapeutic outcomes for αPD-L1 and αPD-1. **a-c**, Mice with MC38 (**a**) or TRAMP-C2 (**b**, **c**) tumors were intravenously injected with 10 μg of αPD-1 (**a**, **b**) or the indicated doses of αPD-1 (**c**) with 20 μg of MC38-EVs (**a**) or TRAMP-C2-EVs (**b**) or without EVs (**c**) every 2 days starting when the tumor size reached 80-100 mm^3. The tumor size was monitored every other day. **d**, Mice with TRAMP-C2 *Rab27a*$^{-/-}$ tumors were intravenously injected with 30 μg αPD-1 or αPD-L1 every 2 days starting when the tumor size reached 80-100 mm^3. The tumor size was monitored every other day. **e**, PD-1$^+$ Treg cells were sorted from *Foxp3*^{GFP} mice and labelled with CellTrace Far Red followed by stimulated with 1 ng ml^{-1} PMA, 200 ng ml $^{-1}$ ionomycin, and 4000 U ml^{-1} murine IL-2 in the presence of 5 μg ml^{-1} recombinant mouse PD-L2 for 72 h. Then, cell proliferation was determined by flow cytometry. Representative results from three independent experiments are shown (n = 5). ***P < 0.001; ns, not significant (unpaired two-tailed Student's t-test; mean and s.d.).

Supplementary Table 1: Information of antibodies used in this article

Antibodies (dilution)	Resource	Identifier
anti-mouse CD9 PE (1:500)	BioLegend	Cat: 124806
anti-mouse CD9 APC (1:500)	BioLegend	Cat: 124812
anti-human CD9 PE (1:500)	BioLegend	Cat: 312105
anti-human CD63 APC (1:500)	BioLegend	Cat: 353007
anti-mouse CD81 APC (1:500)	BioLegend	Cat: 104909
anti-human CD81 APC (1:500)	BioLegend	Cat: 349510
anti-mouse CD274 PE (1:500)	BioLegend	Cat: 124308
anti-mouse CD274 PE (1:500)	BioLegend	Cat: 153612
anti-human CD274 PE (1:500)	BioLegend	Cat: 329706
anti-human CD274 APC (1:500)	BioLegend	Cat: 329708
anti-mouse CD3e (2 μg ml^{-1})	Bio X Cell	Cat: BE0001-1
anti-mouse CD28 (2 μg ml^{-1})	Bio X Cell	Cat: BE0015-5
Mouse IgG1, κ Isotype Antibody APC (1:500)	BioLegend	Cat: 400121
Rat IgG2b, κ Isotype Antibody PE (1:500)	BioLegend	Cat: 400607,
Rat IgG2a, κ Isotype Antibody PE (1:500)	BioLegend	Cat: 400507
Purified anti-mouse CD274 Antibody (1:500)	BioLegend	Cat: 124302
Purified anti-mouse CD279 Antibody (1:500)	BioLegend	Cat: 135202
Purified anti-human CD274 Antibody (1:500)	BioLegend	Cat: 329702
Purified anti-rat IgG2a Antibody (1:500)	BioLegend	Cat: 407502
Biotin anti-rat IgG Antibody (1:500)	BioLegend	Cat: 405428
eBioscience™ CD274 Monoclonal Antibody, Biotin (1:500)	Invitrogen	Cat: 13-5982-82
eBioscience™ Fixable Viability Dye eFluor™ 520 (1:500)	Invitrogen	Cat: 65-0867-14
anti-mouse CD45 PB (1:500)	BioLegend	Cat: 103126
anti-mouse CD44 APC (1:500)	BioLegend	Cat: 103012
anti-mouse CD4 APC/Cyanine7 (1:500)	BioLegend	Cat: 100526
anti-mouse CD8α APC/Cyanine7 (1:500)	BioLegend	Cat: 100714
anti-mouse CD8α APC (1:500)	BioLegend	Cat: 100712
anti-mouse PD-1 PE (1:500)	BioLegend	Cat: 135205
anti-mouse CD62L PE (1:500)	BioLegend	Cat: 161204
anti-mouse IFN-γ PE (1:500)	BioLegend	Cat: 505808
anti-mouse Ki-67 PE (1:500)	BioLegend	Cat: 151209
anti-mouse/human Ki-67 PerCP/Cyanine5.5 (1:500)	BioLegend	Cat: 151221
anti-mouse FOXP3 APC (1:500)	Invitrogen	Cat: 17-5773-82
Mouse anti-CD63 (IF) (1:200)	Invitrogen	Cat: MA1-19281
Rabbit anti-CD63 (WB) (1:1000)	ABclonal	Cat: A5271
Rabbit anti-LAMP1 (IF) (1:200)	Abcam	Cat: ab208943
Rabbit anti-GRP94 (1:1000)	ABclonal	Cat: A0989
Rabbit anti-Tsg101 (1:1000)	ABclonal	Cat: A1692
Rabbit anti-Alix (1:1000)	Proteintech	Cat: 12422-1-AP
Rabbit anti-PD-L1 (IF) (1:200)	Proteintech	Cat: 28076-1-AP
Rabbit anti-PD-L1 (WB) (1:1000)	ABclonal	Cat: A1645

Rabbit anti-Rab27a (1:1000)	ABclonal	Cat: A1934
Rabbit anti-β-Actin (1:1000)	ABclonal	Cat: AC026
Goat anti-mouse IgG HRP (1:200)	MultiSciences	Cat: 70-GAM007
Goat anti-rabbit IgG HRP (1:200)	MultiSciences	Cat: 70-GAR007
Mouse Control IgG (1:500)	ABclonal	Cat: AC011
Rabbit Control IgG (1:500)	ABclonal	Cat: AC005
Purified anti-mouse CD63 Antibody (1:500)	BioLegend	Cat: 143901
Ultra-LEAF™ Purified anti-human CD63 Antibody (1:500)	BioLegend	Cat: 353039
Biotin anti-mouse CD9 Antibody (1:200)	BioLegend	Cat: 124803
Biotin anti-mouse CD81 Antibody (1:200)	BioLegend	Cat: 104903
Biotin anti-mouse F4/80 Antibody (1:200)	BioLegend	Cat: 123105
Goat anti-rabbit IgG DyLight 488 (1:200)	MultiSciences	Cat: 70-GAR4882
Goat anti-mouse IgG DyLight 488 (1:200)	MultiSciences	Cat: 70-GAM4882
Goat anti-mouse IgG DyLight 594 (1:200)	MultiSciences	Cat: 70-GAM5942
Goat anti-rabbit IgG DyLight 594 (1:200)	MultiSciences	Cat: 70-GAR5942

Abbreviations: IF, immunofluorescence; WB, western blotting.

Supplementary Table 2: Information of gRNA sequences used in this article.

Name	Sequence (5' to 3')
Mouse Rab27a gRNA-1	AACCCAGATATAGTGCTGTG
Mouse Rab27a gRNA-2	CCTGAAATCAATGCCCACTG
Mouse Pdl1 gRNA-1	GCCTGCTGTCACTTGCTACG
Mouse Pdl1 gRNA-2	CTTGACGTCTGTGATCTGAA
Mouse Coro1a gRNA-1	ACCAGGCGATGTCTAGCACAGG
Mouse Coro1a gRNA-2	GTAGACAAGAACGTGCCCCTGG
